# Supplementary material for: Intra-Patient Genomic Variations of Human Papillomavirus Type 31 in Cervical Cancer and Precancer
Source: Viruses. 2023 Oct 17;15(10):2104. doi: 10.3390/v15102104 (PMC10612030; doi:10.3390/v15102104)
Supplement: Supplementary file 1 [file viruses-15-02104-s001.zip › viruses-2668267-supplementary.pdf]

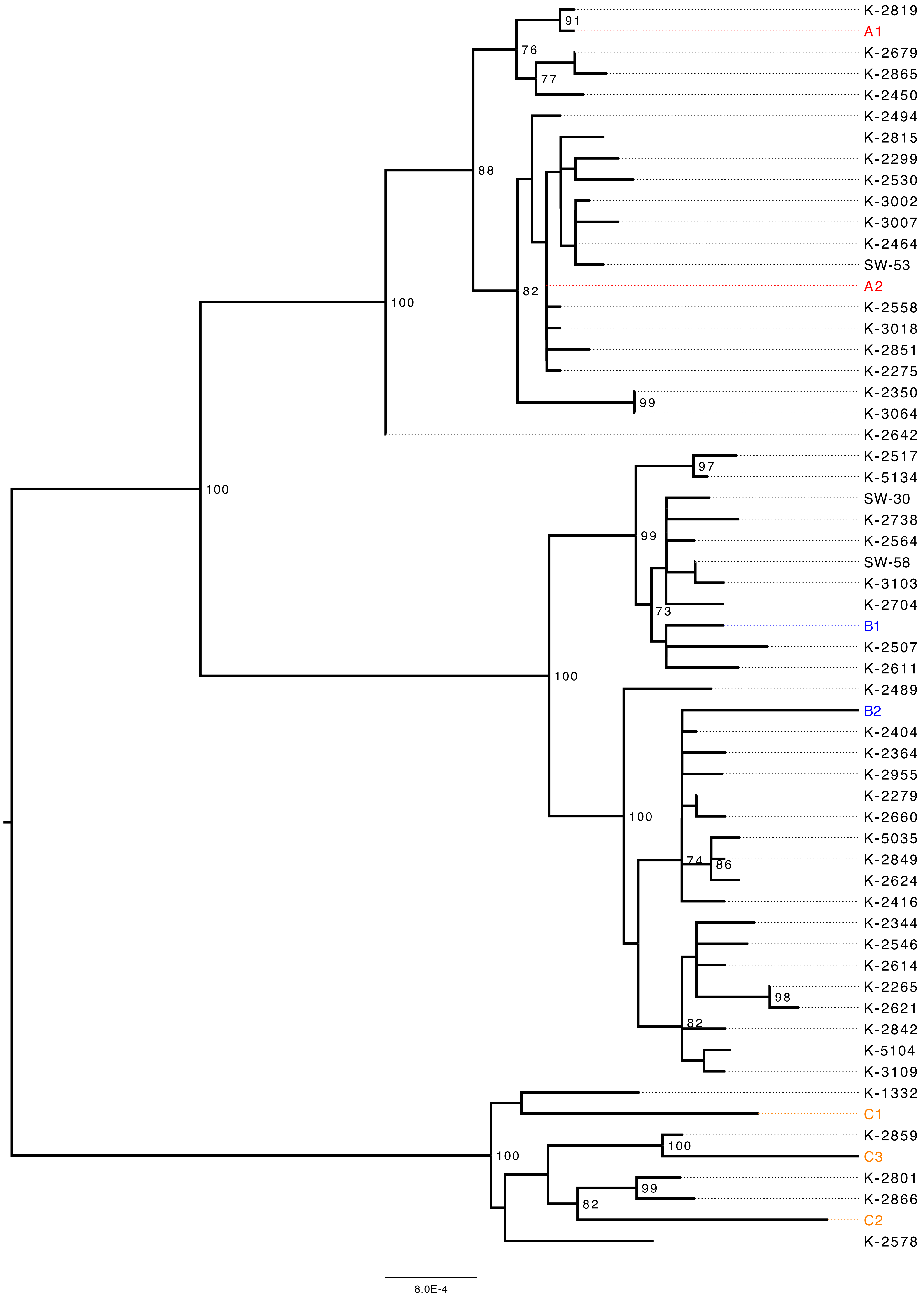

Figure S1. Phylogenetic tree of HPV31 genome sequences of all isolates from Japan (n = 52). Bootstrap values >70% are displayed. Scale bar, nucleotide substitutions per site.
